# Short report: Follow‐up of Bahamian women with a *BRCA1* or *BRCA2* mutation

**DOI:** 10.1002/mgg3.363

**Published:** 2017-12-20

**Authors:** Steven A. Narod, Raleigh Butler, David Bobrowski, Mohammad R. Akbari, DuVaughan Curling, John Lunn, Catherine Ho, Sara Panahi, Marcia Llacuachaqui, Talia Donenberg, Judith Hurley

**Affiliations:** ^1^ Women's College Research Institute Women's College Hospital Toronto ON Canada; ^2^ Dalla Lana School of Public Health University of Toronto Toronto ON Canada; ^3^ Department of Oncology Princess Margaret Hospital Nassau Bahamas; ^4^ Department of Obstetrics and Gynecology Princess Margaret Hospital Nassau Bahamas; ^5^ Department of Hematology and Oncology Doctors Hospital Nassau Bahamas; ^6^ Sylvester Comprehensive Cancer Center University of Miami Miami FL USA

**Keywords:** Bahamas, *BRCA1*, *BRCA2*, breast cancer, genetic testing, prophylactic bilateral mastectomy, prophylactic bilateral salpingo‐oophorectomy

## Abstract

**Purpose:**

We sought to determine to what extent the knowledge of carrying a *BRCA1* or *BRCA2* mutation influences the uptake of preventive surgeries in Bahamian women, including bilateral salpingo‐oophorectomy and bilateral mastectomy.

**Patients and methods:**

The study population consisted of 78 female residents of the Bahamas for whom a *BRCA1* or *BRCA2* mutation had been detected between 2004 and 2014. The mean age of the 78 participants at the time of genetic testing was 46 years (age range 22–73 years). The mean time of follow‐up was 4.4 years.

**Results:**

Of the 78 study participants, 19 women had a bilateral salpingo‐oophorectomy (24%). Seven out of 37 patients who had unilateral breast cancer chose to remove the unaffected contralateral breast (19%). Three of 13 patients with no history of breast cancer chose to have a prophylactic bilateral mastectomy (23%).

**Conclusion:**

Preventive surgery is an acceptable option for a significant proportion of Bahamian women with a *BRCA1* or *BRCA2* mutation. It will be important to identify and reduce barriers to preventive surgery in the Bahamas in order that the benefit of getting testing can be fully realized.

## 1 INTRODUCTION

The Bahamas is an island country in the Caribbean with a population of 300,000 which has approximately 100 new breast cancer cases per year (Akbari et al., 2014). The Bahamas has the highest known prevalence of BRCA mutations among breast cancer patients of any country; 23% of women with breast cancer in the Bahamas were found to carry one of seven founder mutations in the *BRCA1* or *BRCA2* gene (Akbari et al., 2014; Trottier et al., 2016). From 2004 to 2008 selected familial cases of breast cancer were offered testing. In 2012, we began, on a research basis, genetic testing of all Bahamian women diagnosed with breast cancer (Trottier et al., 2015, 2016). In the event of a positive test result, unaffected female relatives were offered testing. The benefit of genetic testing is derived in part from preventive surgery and therefore it is important to assess the numbers of preventive surgeries performed if we wish to evaluate the efficacy of the Bahamas breast cancer genetic testing initiative. Risk‐reducing surgical options include prophylactic bilateral salpingo‐oophorectomy and bilateral mastectomy. Oophorectomy is recommended to women with and without breast cancer. Women with a prior history of breast cancer and an intact contralateral breast may opt for contralateral mastectomy. Women with no previous history of breast cancer may opt for bilateral mastectomy. Prophylactic bilateral mastectomy has been reported to reduce the risk of breast cancer by approximately 95% (Rebbeck, 2004). Prophylactic contralateral mastectomy has been shown to reduce the risk of death from breast cancer in *BRCA1* and *BRCA2* carriers by approximately 50% (Metcalfe et al., 2014). Prophylactic bilateral salpingo‐oophorectomy has been associated with an 80% reduction in the risk of ovarian, fallopian tube, or peritoneal cancer (Finch et al., 2014) and with a 70% reduction in all‐cause mortality. Women in the Bahamas with a *BRCA1* or *BRCA2* mutation are seen in person by a genetic counselor for test disclosure and are counseled with regard to the options available to them. They are offered follow‐up appointments with a breast surgeon and with a gynecology oncologist and preventive surgeries are covered under the national health plan. MRI screening of the breast is not available. We surveyed all women who were identified to have a positive genetic test about preventive cancer surgeries. The results of this survey will be useful in evaluating the health outcomes associated with genetic screening and for planning genetic services for BRCA mutation carriers in the Bahamas.

## 2 METHODS

### 2.1 Study population

The survey population included female residents in the Bahamas for whom a mutation had been detected by genetic testing through the course of several studies conducted between 2004 and 2012 (Akbari et al., 2014; Trottier et al., 2015, 2016). When a mutation was identified in a family, genetic counseling and testing was offered to other at‐risk women in the family (Trottier et al., 2015). Women were recruited to these studies from public and private sources, including clinics in Freeport and Nassau in the Bahamas as described. For the purposes of this study, all carriers of *BRCA1* and *BRCA2* mutations identified to date were enrolled.

A total of 107 *BRCA1* and *BRCA2* mutation carriers were identified and registered in the study database. If the study subject was deceased or could not be reached after three or more attempts, the next‐of‐kin, usually defined as a sister or daughter of the mutation carrier, was contacted. We were able to obtain relevant information from 16 deceased women through their next of kin. We were unable to contact 26 of 107 mutation carriers and these were excluded from the study. Three other women were excluded because of missing information. The remaining 78 participants were registered in the study (62 were alive and 16 were deceased). This study was approved by the ethics board at Women's College Hospital, Toronto, Canada.

### 2.2 Study protocol

From May 2016 to August 2016, members of the investigator team contacted 78 *BRCA1* and *BRCA2* mutation carriers by telephone. The telephone interview consisted of the completion of a standard follow‐up questionnaire; relevant study data included the participant's age, family history, cancer history, and the date the genetic test result was received. The questionnaire also included questions related to screening history and cancer preventive procedures, including bilateral salpingo‐oophorectomy and prophylactic bilateral mastectomy.

## 3 RESULTS

### 3.1 Demographics

Seventy‐eight women were identified as having a *BRCA1* or *BRCA2* mutation through the study database and were interviewed for the study. The mean age of the 78 participants at the time of genetic testing was 46 years (age range 22–73 years) (Table 1) and at the time of questionnaire completion was 50 years. Of these, 73 women (94%) had a *BRCA1* mutation and 5 women (6%) had a *BRCA2* mutation (Table 2).

**Table 1:** Distribution of mutation carriers by age at genetic testing

Age breakdown (range)	Number of participants
20–29	8
30–39	16
40–49	28
50–59	12
60+	14
Total	78

**Table 2:** Distribution of mutations for 78 study participants

Gene	Exon	Confirmed mutation	Number of subjects	Percentage of subjects with mutation
*BRCA1*		IVS13 + 1G>A	42	54
*BRCA1*	15	4730insG	10	13
*BRCA1*	21	T5443G	9	11
*BRCA1*		IVS16 + 6T>C	4	5
*BRCA1*	11	943ins10	3	4
*BRCA1*	2	185delAG	3	4
*BRCA1*	11	3477delGT	1	1
*BRCA1*	8 and 9	Deletion	1	1
*BRCA2*	17	8128delA	5	6

Sixteen of the 78 women (21%) died in the follow‐up period; 12 deaths were due to breast cancer (15%), two were due to ovarian cancer (3%), and one each were attributed to kidney failure and heart failure. Of the 62 survivors, 47 patients (76%) reported a diagnosis of breast cancer prior to receiving the genetic test result and four reported a diagnosis of ovarian cancer prior to the genetic test result. Of the 15 women without either cancer at the time of diagnosis, there were two new cases of breast cancer in the follow‐up period (annual incidence 3.0%).

### 3.2 Uptake of carrier risk reduction options

Five women had a bilateral oophorectomy prior to receiving a genetic test result. Of the remaining 55 women with intact ovaries at the time of genetic testing, 16 had a bilateral oophorectomy after the genetic test result was disclosed, including 14 of 42 women with breast cancer (29%) and 2 of 13 women (15%) without breast cancer.

Forty‐six women had diagnosis of breast cancer prior to the genetic test result and two were diagnosed with breast cancer in the follow‐up period. Fifteen subjects had no diagnosis of breast or ovarian cancer at the time of the genetic test result.

Thirty‐five of the breast cancer patients had a unilateral mastectomy and 11 had a bilateral mastectomy as initial treatment (prior to knowledge of their positive mutation status).

Of the 35 patients with a breast cancer diagnosis prior to genetic testing and an intact contralateral breast 6 (17%) opted for removal of the unaffected contralateral breast (19%). Following genetic testing, 3 out of 13 women without a personal history of breast or ovarian cancer had a prophylactic bilateral mastectomy (23%).

Three women had both breast and ovarian cancer prior to genetic testing and none of these chose to pursue any further intervention.

## 4 DISCUSSION

In this study, we followed 78 Bahamian women who had been told that they carried a mutation in *BRCA1* or *BRCA2*. The majority of these women had been tested because they had breast cancer but 15 women (mostly relatives of carriers) were tested when unaffected. The original testing program was modest in scale and the 78 mutation carriers was the yield of the testing of approximately 75 familial cases and 225 unselected breast cancer patients, followed by testing unaffected relatives of known carriers. The efficiency of this approach is due to the high prevalence of mutations among breast cancer patients and the ability to screen for founder mutations. Also, testing was done without cost to the patients. It is expected that in the near future, inexpensive next generation sequencing will be a universal standard and will replace screening with panels of variant founder alleles. For example, in Canada, universal genetic testing for *BRCA1* and *BRCA2* is now available to all Canadian women based on next generation sequencing (http://www.thescreenproject.ca) and we hope to develop a similar national program to the Bahamas as well in the next year.

We used the number of preventive surgeries performed as a measure of success of our genetic testing initiative. Among the 78 study participants, 19 oophorectomies, 14 contralateral mastectomies, and 3 bilateral mastectomies were performed. These counts are perhaps lower than ideal but are encouraging that our genetic testing is fruitful with a relatively modest investment and our analysis supports the continuation of these efforts. It has been suggested that the protective effect of surgery makes hereditary breast and ovarian cancer to a large extent a preventable disease (Domchek, 2010). Finch et al. (2014) have shown that prophylactic bilateral salpingo‐oophorectomy in mutation carriers is associated with a 70% reduction in all‐cause mortality until age 70. A prior report of risk reduction options in Canadian women with BRCA mutation documented a rate of prophylactic bilateral mastectomy uptake at 36% and an uptake of prophylactic bilateral salpingo‐oophorectomy of 61% (Metcalfe et al., 2012). Metcalfe et al. (2012) reported on a 2 year follow‐up of Jewish women with *BRCA1* and *BRCA2* mutation who underwent population genetic screening. 90% of the carriers identified accepted prophylactic bilateral salpingo‐oophorectomy, however, only 11% chose prophylactic bilateral mastectomy (similar to the proportion observed in the Bahamas.) Madalinska et al. (2007) reported that almost three quarters of *BRCA1/2* mutation carriers in the Netherlands undergo prophylactic bilateral salpingo‐oophorectomy. Evans et al. (2009) reported that in the United Kingdom, the rate of prophylactic bilateral salpingo‐oophorectomy was approcimatey 60% for *BRCA1* carriers and 40% for *BRCA2* carriers. However, based on the age‐specific incidence rates of breast and ovarian cancer, we recommend bilateral oophorectomy at age 35 in *BRCA1* carriers and at age 40 in *BRCA2* carriers.

Several factors may contribute to the relative low rates of preventive surgery in the Bahamas, such as a lack of knowledge regarding the risks of cancer, fear of cancer and access to surgery. All patients were counseled by a highly trained genetic counselor who informed them of the pros and cons of risk‐reducing surgery. The rate of preventive mastectomy is low. It is not clear if the barriers to preventive surgery are financial, are due to access to care or are social/psychological and we think that the identification of possible barriers should be the topic of a further study. Physicians involved in all phases of patient care should engage their *BRCA1* and *BRCA2* positive patients in the decision‐making process and should discuss the benefits of salpingo‐oophorectomy and prophylactic bilateral mastectomy.

## CONFLICT OF INTEREST

None declared.
